# A Survey of Long Case Psychotherapy Experiences of Psychiatric Trainees Working in South London and Maudsley NHS Trust

**DOI:** 10.1192/bjo.2022.105

**Published:** 2022-06-20

**Authors:** Famia Askari, Gillian Brown

**Affiliations:** South London and Maudsley NHS Foundation Trust, London, United Kingdom

## Abstract

**Aims:**

Psychotherapy is a mandatory component of the Royal College of Psychiatrists training curriculum. The long-term benefits of psychotherapeutically-informed practice to both patients and doctors are well recognised. In the face of regular service configurations, there was a wish to gather evidence to ensure continued provision of this training experience to a high quality. The purpose of this survey was to obtain feedback from trainees regarding their experience of the psychodynamic psychotherapy long case to evidence the relevance and value of this component of the training programme.

**Methods:**

The anonymous survey, including questions, numerical rating scales and free text boxes, was sent to 294 trainees on a combined mailing list. This number may be slightly inaccurate due to incorrect email addresses and duplicates. A reminder email was sent one week later to encourage a higher fill rate.

**Results:**

There were 35 responses: a fill rate of approximately 12%. The largest group of respondents were Core Trainees (3rd year) of whom just over half had completed the long case.

92% of respondents found the long case to be at least ‘slightly useful’, of whom almost a third found it ‘extremely useful’. 94% of trainees found the experience to be at least ‘slightly helpful’ in understanding psychodynamic concepts and 75% found supervision ‘very’ or ‘extremely useful’.

Qualitative responses described it as a unique experience not offered elsewhere in the curriculum that provided important transferable skills.

Difficulties mentioned were similar to those found by previous studies, including practical concerns relating to patient and service factors. There were suggestions for more in-depth training and suggested reading to increase trainees’ confidence. An email was sent signposting trainees to further support in response to some specific concerns.

**Conclusion:**

Overall, the responses suggest that the majority of trainees find the long case a valuable training opportunity. These data are useful to evaluate and improve trainees’ experience within the trust, and could be helpful for other training programmes nationally.

Due to the nature of psychotherapy, there is inevitable variation in trainee experiences but attempts to clarify and/or standardise some elements may result in greater trainee satisfaction. Trainees suggested improvements including addressing practical issues, patient factors, supervision content, and educational resources. A future survey following implementation of some suggested improvements would be helpful; the impact of the COVID-19 pandemic and the switch to remote working is another area that may be useful to explore.

